# Circadian Intraocular Pressure Profiles in Chronic Open Angle Glaucomas

**Published:** 2010-04

**Authors:** Jost B Jonas, Wido M Budde, Andrea Stroux, Isabel M Oberacher-Velten, Anselm G Juenemann

**Affiliations:** 1 Department of Ophthalmology, Faculty of Clinical Medicine, University of Heidelberg, Mannheim, Germany; 2 Department of Ophthalmology, University Erlangen-Nürnberg, Erlangen, Germany; 3 Department of Medical Informatics, Biometry and Epidemiology, Campus Benjamin Franklin, Charité Berlin, Hindenburgdamm, Berlin, Germany

**Keywords:** Intraocular Pressure, Amplitude, Profile, Fluctuation, Chronic Open Angle Glaucoma, Central Corneal Thickness

## Abstract

**Purpose:**

To evaluate circadian intraocular pressure (IOP) profiles in eyes with different types of chronic open-angle glaucoma (COAG) and normal eyes.

**Methods:**

This study included 3,561 circadian IOP profiles obtained from 1,408 eyes of 720 Caucasian individuals including glaucoma patients under topical treatment (1,072 eyes) and normal subjects (336 eyes). IOP profiles were obtained by Goldmann applanation tonometry and included measurements at 7 am, noon, 5 pm, 9 pm, and midnight.

**Results:**

Fluctuations of circadian IOP in the secondary open-angle glaucoma (SOAG) group (6.96±3.69 mmHg) was significantly (P<0.001) higher than that of the normal pressure glaucoma group (4.89±1.99 mmHg) and normal eyes (4.69±1.95 mmHg); but the difference between the two latter groups was not significant (P=0.47). Expressed as percentages, IOP fluctuations did not vary significantly among any of the study groups. Inter-ocular IOP difference for any measurement was significantly (P<0.001) smaller than the profile fluctuations. In all study groups except the SOAG group, IOP was highest at 7 am, followed by noon, 5 pm, and finally 9 pm or midnight. In the SOAG group, mean IOP measurements did not vary significantly during day and night.

**Conclusions:**

In contrast to normal eyes and eyes with primary open-angle glaucoma under topical antiglaucoma treatment, eyes with SOAG under topical treatment do not show the usual circadian IOP profile in which the highest IOP values occur in the morning, and the lowest in the evening or at midnight. These findings may have implications for timing of tonometry. Fluctuation of circadian IOP was highest in SOAG compared to other types of open angle glaucomas.

## INTRODUCTION

Intraocular pressure (IOP) is one of the most important risk factors for development and progression of glaucomatous optic neuropathy.^1^–^3^ Studies by Becker, Phelps and other investigators have revealed that IOP shows marked diurnal and nocturnal variability.^4^–^33^ This intra-individual 24-hour variability in IOP may be important both clinically and pathogenetically. From a diagnostic point of view, a single IOP measurement may miss the maximal IOP and thus lead to false assumptions regarding the adequacy of IOP control. Diurnal variation of IOP may pathogenetically be important if different types of chronic open-angle glaucoma (COAG) vary in the amplitude of IOP fluctuation. Large fluctuations of IOP have been suggested to be a risk factor for progression of glaucoma.^34^,^35^

Information on variations of IOP profiles in different types of COAG under treatment has been scarce, therefore we designed this study to evaluate whether circadian IOP measurements differ in normal eyes and eyes with COAG under treatment; whether the IOP profile and its fluctuation differ in primary open-angle glaucoma (POAG) and secondary COAG; and whether intra-individual inter-ocular circadian differences in IOP vary in normal subjects and patients with glaucoma.

## METHODS

This clinical observational study included 3,561 circadian IOP profiles measured on 1,408 eyes (704 right eyes) of 720 Caucasian individuals including 336 normal eyes, 398 eyes with ocular hypertension, 278 eyes with pre-perimetric glaucoma, 140 eyes with primary open-angle glaucoma, 46 eyes with secondary open-angle glaucoma, and 210 eyes with normal-pressure glaucoma. Normal subjects were members of the hospital administration without any sign of optic nerve disease including glaucoma. Follow-up examinations were performed, during which 863 eyes were assessed for a second circadian IOP profile, and 532 eyes for a third. All individuals had best-corrected visual acuity of 20/25 or better. Glaucoma patients were under treatment with topical antiglaucoma medications.

Exclusion criteria were ocular diseases other than glaucoma and presence of diabetes mellitus. Informed consent was obtained from each subject before enrolment. Institutional Review Board/Ethics Committee approval was obtained at the start of the study.

The number of IOP profiles per subject depended on the time of enrolment of the subject and on the subject’s availability. IOP profiles were scheduled once to twice per year per subject. For all eyes included in the study, at least one circadian IOP profile was obtained including measurements at 7 am, noon, 5 pm, 9 pm and midnight. For night measurements, the patients either stayed awake till midnight, or were awakened and maintained upright position for at least 15 minutes before tonometry. IOP was measured using slitlamp mounted Goldmann applanation tonometers by experienced residents in the university hospital. High IOP was defined as IOP>21 mmHg. Amplitude of fluctuations was defined as the difference between the highest and lowest IOP readings.

The COAG group included eyes with POAG, secondary open angle glaucoma (SOAG), ocular hypertension (OHT), and normal pressure glaucoma (NPG). SOAG was due to pseudoexfoliation or pigment dispersion syndrome. Criteria for the diagnosis of NPG were maximal IOP readings of 21 mmHg or less in at least two 24-hour pressure profiles. Ocular hypertension was defined as IOP measurements higher than 21 mmHg without visual field defects and without glaucomatous abnormalities of the optic nerve head. Pre-perimetric glaucoma was defined by glaucomatous abnormalities of the optic nerve head but normal white-on-white visual fields. Perimetric glaucoma was defined by glaucomatous abnormalities of the optic nerve head and glaucomatous visual field defects.

A glaucomatous visual field defect was defined as an Octopus G1 field with (a) at least three adjacent test points having a deviation of equal to or greater than 5 dB and one test point with a deviation greater than 10 dB lower, (b) at least two adjacent test points with a deviation equal to or greater than 10 dB, (c) at least three adjacent test points with a deviation equal to or greater than 5 dB abutting the nasal horizontal meridian, or (d) a mean visual field defect of more than 2 dB. Rate of false positive and false negative responses had to be less than or equal to 15%. Two visual field tests were performed prior to inclusion into the study to obtain baseline data. Glaucomatous changes of the optic nerve head included an unusually small neuroretinal rim area in relation to the optic disc size, abnormal shape of the neuroretinal rim, greater vertical than horizontal cup to disc ratio, and localized or diffuse retinal nerve fibre layer defects.^36^

The patients were receiving routine ophthalmic care including topical antiglaucoma medications. There was no major difference in the type of treatment between the various glaucoma subgroups with most of the eyes receiving topical beta-blockers twice daily and a prostaglandin analogue in the evening.

Statistical analysis was performed using a commercially available statistical software package (SPSS for Windows, version 11.5, SPSS, Chicago, USA). Two-way analysis of variance was applied to investigate differences in circadian IOP fluctuations. Dependency of left and right eyes from the same subject was taken into account conservatively: Chi-square values were multiplied by the factor “number of patients divided by number of eyes”. Thus significance tests were performed with respect to the number of patients rather than the number of eyes.

## RESULTS

Mean patient age was 48.7±12.2 (range, 12–76) years and mean refractive error was −0.92±2.40 (range, −12.75 to +7.0) diopters. During the first IOP profile (n=1,408), the number of topical agents for each patient was one in 442 (31.4%), two in 168 (11.9%), three in 59 (4.2%), and four in 8 (0.6%) eyes. For 731 (51.9%) eyes including 313 normal eyes, 152 eyes with ocular hypertension, 104 eyes with pre-perimetric glaucoma, 38 eyes with primary open-angle glaucoma, 115 eyes with normal-pressure glaucoma, and 9 eyes with secondary open-angle glaucoma, no medication was given.

**Figure 1:**
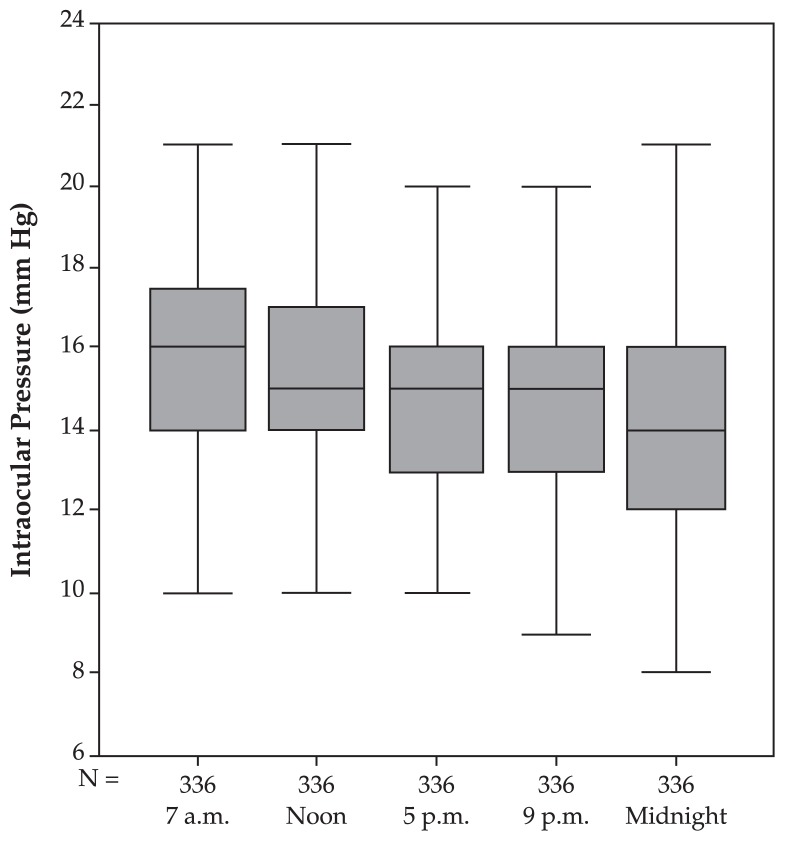
Intraocular pressure profile (mean and standard deviation) in the normal group.

In normal eyes, mean fluctuation of circadian IOP was 4.69±1.95 mmHg. Mean IOP at 7 am was highest and was significantly (P=0.017) higher than measurements taken at noon, slightly higher (P=0.079) than measurements taken at 5 pm, not significantly (P=0.204) higher than measurements taken at 9 pm, and significantly (P<0.001) higher than measurements taken at midnight (Table 1, Fig. 1). Two-way analysis of variance with respect to polynomial contrasts confirmed linearity of the circadian profile curve (P<0.001).

**Figure 2:**
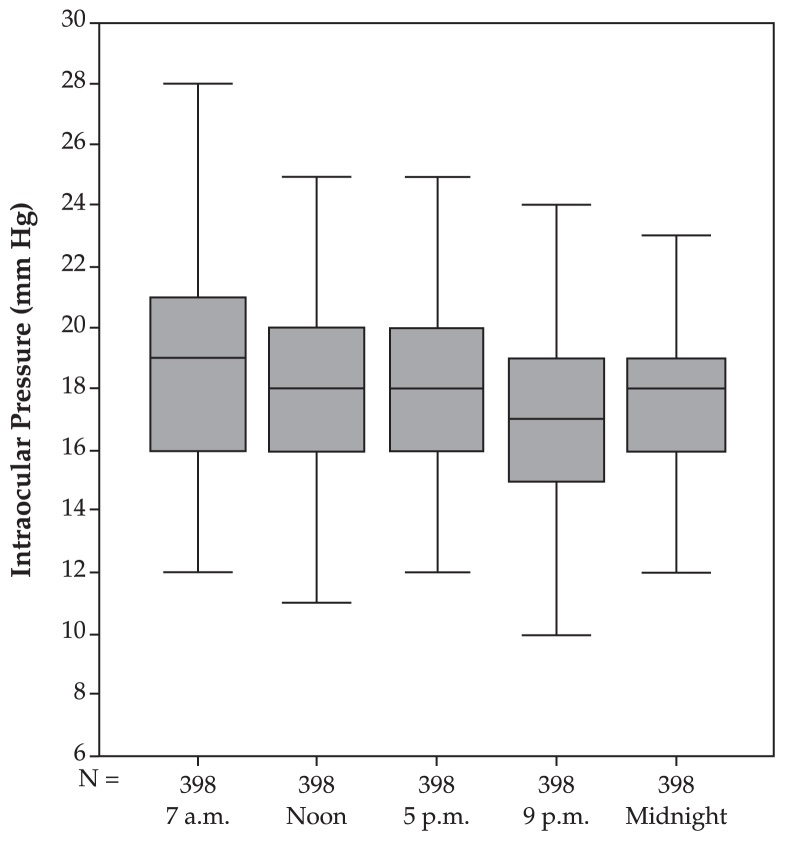
Intraocular pressure profile (mean and standard deviation) in the ocular hypertensive group.

In ocular hypertensive eyes, mean fluctuation of circadian IOP was 6.00±3.14 mmHg which was significantly (P<0.001) higher than normal eyes. IOP at 7 am was the highest and was significantly (P<0.001) higher than measurements taken at noon, not significantly (P=0.72) different from measurements taken at 5 pm, significantly (P<0.001) higher than 9 pm, but not markedly (P=0.16) different from midnight (Table 1, Fig. 2). The circadian IOP profile showed significant linearity (P<0.001).

**Figure 3:**
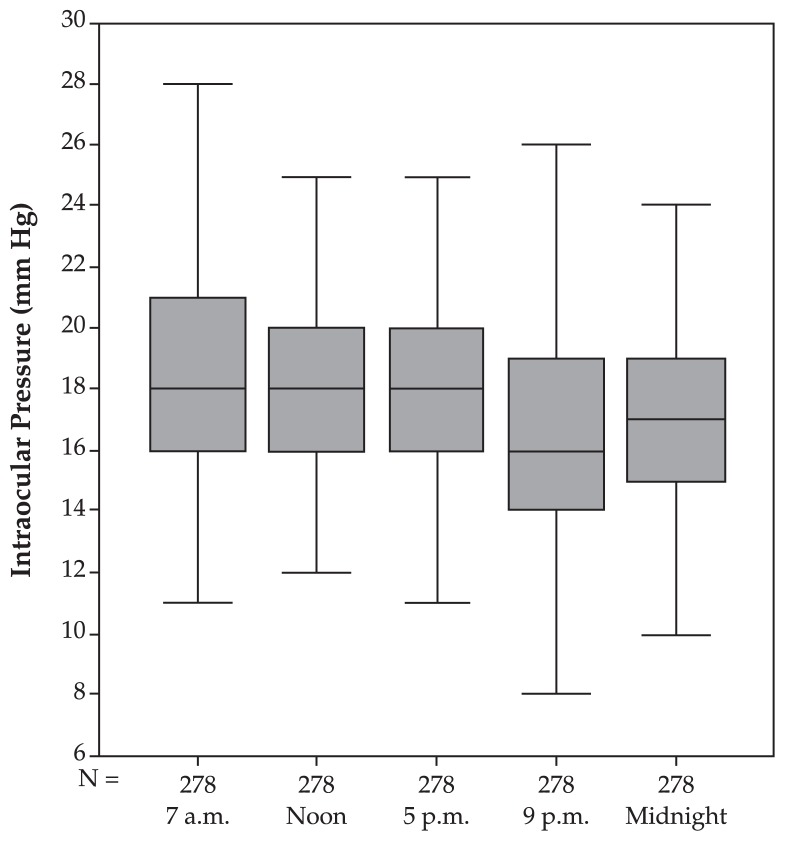
Intraocular pressure profile (mean and standard deviation) in the pre-perimetric glaucoma group.

In the pre-perimetric glaucoma group, IOP measurements decreased linearly towards midnight (P<0.001). Mean IOP at 7 am was the highest, which was not significantly (P=0.08) higher than measurements taken at noon, not significantly (P=0.14) higher than 5 pm, significantly (P<0.001) higher than measurements taken at 9 pm but not significantly (P=0.16) different from midnight. Mean fluctuation of circadian IOP was 6.00±2.96 (median, 5; range, 1–30) mmHg which was significantly (P<0.001) higher than that of the normal group but not significantly (P=0.94) different from the ocular hypertensive group (Table 1, Fig. 3).

**Figure 4:**
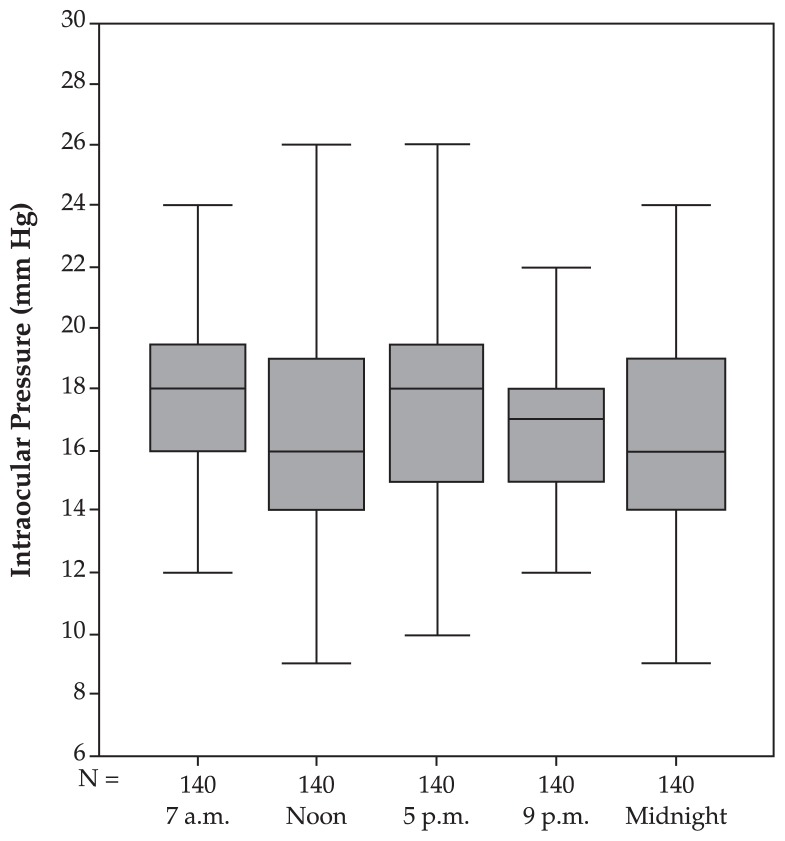
Intraocular pressure profile (mean and standard deviation) in the primary open-angle glaucoma group.

In the perimetric POAG group, IOP measurements taken at 7 am, noon, and 5 pm did not differ significantly (P>0.05) from one other; even though measurements taken at 7 am and 5 pm were slightly, however non-significantly, higher than 9 pm (P=0.10) and midnight (P=0.074). No significant linearity of the IOP curve with the time of measurement could be observed (P=0.13). Combining eyes with pre-perimetric glaucoma with perimetric POAG, the IOP measurements decreased significantly with daytime. Measurements at midnight were the smallest (P<0.01). Mean fluctuation of circadian IOP was 5.74±3.44 (median, 5; range 1–21) mmHg which was not significantly different from the normal group (P=0.067), the pre-perimetric glaucoma group (P=0.22) or the ocular hypertensive group (P=0.246) (Table 1, Fig. 4).

**Figure 5:**
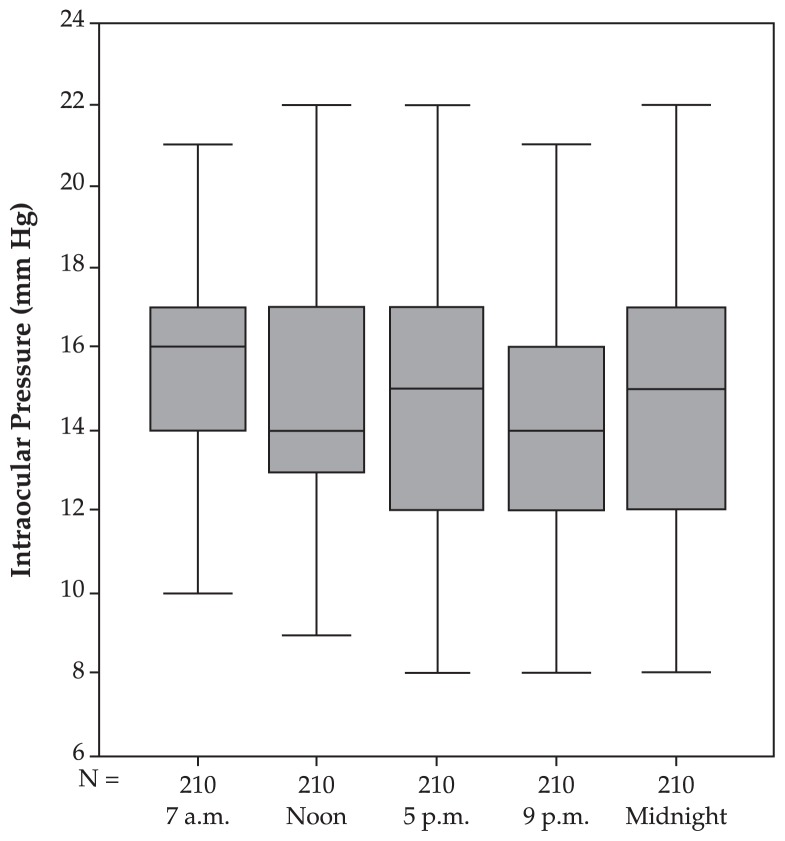
Intraocular pressure profile (mean and standard deviation) in the normal-pressure glaucoma group.

In the normal pressure glaucoma group, mean fluctuation of circadian IOP was 4.89±1.99 mmHg which was not significantly different from that of normal eyes (P=0.48) and the POAG group (P=0.24), but significantly lower than ocular hypertensive eyes (P<0.001) and the pre-perimetric glaucoma group (P<0.001). The profile showed a linear decrease towards midnight (P=0.03). Mean IOP at 7 am was non-significantly (P=0.077) higher than noon and showed a non-significant decrease (P=0.73) towards 5 pm. Mean IOP at 9 pm was non-significantly (P=0.18) lower than 5 pm and non-significantly (P=0.42) different from IOP at midnight (Table 1, Fig. 5).

**Figure 6:**
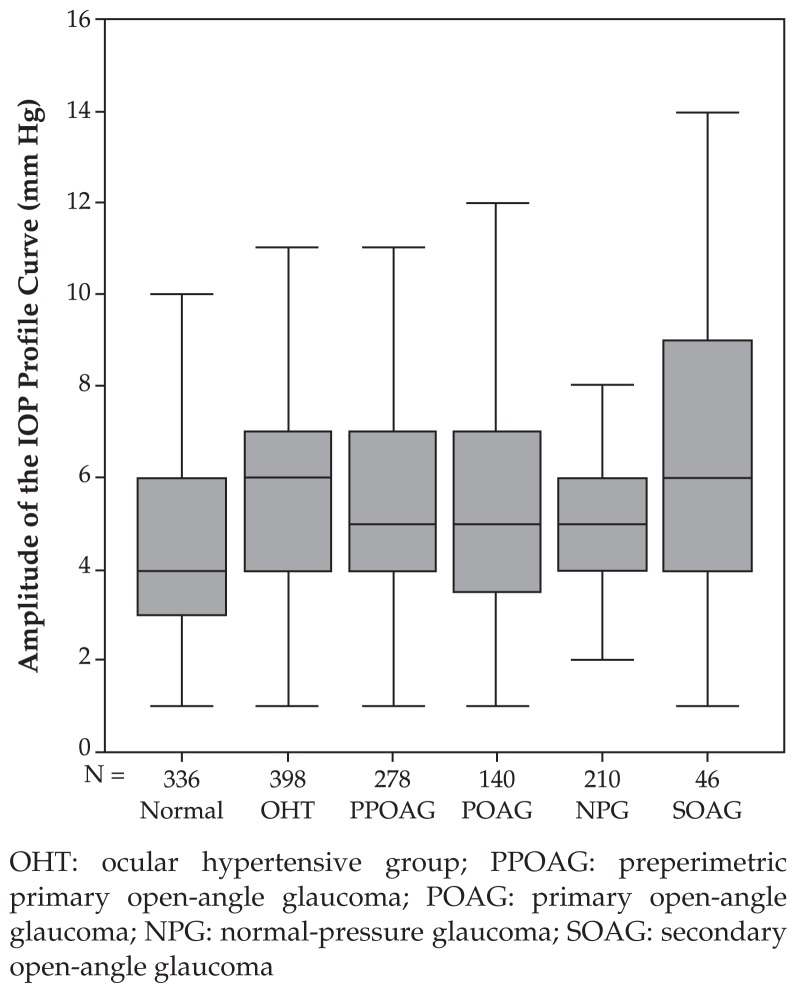
Boxplots showing the amplitude of intraocular pressure profiles in the various study groups. The pressure amplitude in the secondary open-angle glaucoma group was significantly higher than in the normal group (P<0.001) and in the normal-pressure glaucoma group (P<0.001). It did not vary significantly from the pressure amplitude measured in the ocular hypertensive group (P=0.192), the primary open-angle glaucoma group (P=0.072), and the preperimetric glaucoma group (P=0.191). OHT: ocular hypertensive group; PPOAG: preperimetric primary open-angle glaucoma; POAG: primary open-angle glaucoma; NPG: normal-pressure glaucoma; SOAG: secondary open-angle glaucoma

In the secondary open-angle glaucoma group, no linear decrease in diurnal IOP profiles could be observed. Mean IOP at 7 am was not significantly different from that of noon (P=0.65) and 5 pm (P=0.54). Mean IOP at 5 pm was significantly (P=0.026) higher than that of 9 pm but not significantly (P=0.73) different from midnight. Mean fluctuation of circadian IOP was 6.96±3.69 mmHg which was significantly higher than normal eyes (P<0.001) and the normal pressure glaucoma group (P<0.001) but not significantly different from ocular hypertensive eyes (P=0.19), POAG eyes (P=0.072), and the pre-perimetric glaucoma group (P=0.19) (Table 1, Fig. 6).

Within all glaucoma subgroups, as well as in normal eyes, fluctuation of circadian IOP was independent of age, gender, laterality of the eye, refractive error, central corneal thickness, and anterior corneal curvature (data not presented). Expressed as percentages of IOP value, mean IOP did not vary significantly between any of the study groups (data not presented).

The number of IOP profiles for each eye is shown in Table 2. The above-mentioned results were confirmed when repeating circadian profiles a second, third or fourth time (data not presented). During 1,303 circadian IOP profiles, the patients were under monotherapy with topical anti-glaucoma medications (Table 3).

Regarding intraindividual inter-ocular differences, IOP rise during circadian IOP profiles usually occurred at the same time in both eyes. Absolute inter-ocular IOP differences between the right and left eye for any measurement time was significantly (P<0.001) smaller than the circadian IOP fluctuation. Inter-ocular differences in IOP were independent of age, refractive errors, gender and optic disc size (P>0.05). Absolute inter-ocular IOP differences at different measurement times did not differ from each other (P=0.27) (data not presented).

## DISCUSSION

It is well documented that IOP fluctuates at different times of day and night which has prompted diurnal and circadian IOP measurements.^1^–^35^ In the present study IOP was highest at 7 am in all subgroups except for secondary open-angle glaucomas, followed by measurements at noon, 5 pm, and finally by measurements at 9 pm or at midnight. In the secondary open-angle glaucoma group, mean measurements did not vary significantly during day and night. Mean fluctuation of circadian IOP was 5.5±2.8 mmHg, overall. The amplitude of fluctuations was highest in the secondary open-angle glaucoma group as compared to other study groups, but it did not differ significantly between the normal pressure glaucoma group and the normal group. The inter-ocular IOP difference for any time of measurement was significantly (P<0.001) smaller than the amplitude of IOP fluctuations.

The present study agrees and partially disagrees with previous studies. Sacca et al^13^ found that the highest IOP values were detectable in the morning in normal subjects, patients with POAG and those with normal pressure glaucoma; the lowest values were found in the early afternoon hours. In a large study by David and coworkers^8^ evaluating 690 diurnal curves, the highest IOP was detected during the earliest morning measurement in 40% of the patients. The lowest IOP measurement showed no specific predilection for any particular time of the day. As in our study, David and coworkers found a mean range of IOP fluctuation during diurnal curves of 5.0 mmHg in normal subjects, 5.8 mmHg in patients with open-angle glaucoma, and 6.8 mmHg in patients with ocular hypertension. As in the present investigation, David et al did not find any association between diurnal IOP fluctuation and the patients’ diagnosis, age and gender. Finding a peak in the IOP curves in the early morning also confirms a previous investigation by Wilensky and coworkers.^9^ In their study, diurnal IOP curves obtained by home tonometry showed a morning or mid-day maxima. In our study, patients with secondary open-angle glaucoma had higher IOP fluctuation than patients with primary open-angle glaucoma or normal subjects which is consistent with studies by Konstas and coworkers, and others.^12^,^13^

The findings of the present study as well as previous studies on circadian IOP curves may have clinical applications. In normal eyes as well as eyes with primary open-angle glaucoma, IOP measurements decrease with the time of the day; this may suggest that if a patient is always examined late in the afternoon, one may overlook higher IOP values and the patient may erroneously be regarded to have “normal pressure glaucoma”. Similar discussions have already been made by Sacca et al and others.^13^,^17^ The diurnal variation of IOP may have implications in follow-up of patients with glaucoma. If tonometry during follow-up examinations reveals a different IOP than the previous examination, it does not necessarily reflect failure or success of treatment. Such a finding may be due to differences in the time when IOP measurements are taken.

Potential limitations of our study should be mentioned. Firstly, IOP measurements depend on corneal thickness^37^ which may decrease during daytime and thereby artificially decrease IOP. However we only included eyes that had clear corneas and BCVA of 20/25 or better. Secondly, IOP measurements at midnight were obtained in sitting position at the slitlamp and not with the patients lying supine. Information obtained in the present study can therefore only be transferred to the period of time between 7 am and midnight, the time when many patients usually go to bed. The dependence of IOP on body position (sitting versus supine) also limits the comparability of the results of our study with findings reported by other investigations which measured IOP in supine position during sleep.^10^,^15^,^18^ Thirdly, our results may be valid only for eyes without previous filtering surgery. In a recent study by Medeiros and colleagues,^16^ IOP peak and its fluctuation during diurnal pressure curves were significantly greater in a group of glaucoma patients under topical medical therapy than patients who had undergone filtering surgery. Fourthly, IOP was highest at 7 am and lowest at 9 pm/midnight. Since most of the medications had a twice-daily dosing, this IOP pattern could simply be due to the fact that 7 am readings were taken just before the morning dose, while the 9 pm/midnight readings were taken 2 to 4 hours after the evening dose when the drugs were exerting their maximal effect. In addition, all IOP lowering drugs do not function similarly and have different effects on the circadian IOP profile. The resulting IOP profiles were therefore, influenced by the combined effects of treatment, patient compliance, disease and physiologic changes in IOP.

Despite these limitations, the results show when peaks and troughs of IOP profiles in patients under antiglaucomatous topical treatment are to be expected. Correspondingly, the study shows that in the usual clinical setting, it is important to measure IOP at different times of the day, especially in patients with normal pressure glaucoma.

In summary, in contrast to normal eyes and eyes with primary open-angle glaucoma under topical antiglaucoma treatment, eyes with secondary open-angle glaucoma (pseudoexfoliation and pigment dispersion) under topical treatment do not show the usual circadian IOP profile with the highest IOP values in the morning and the lowest in the evening or at midnight. This may have implications for timing of tonometry. We observed that under topical antiglaucoma treatment, eyes with primary open-angle glaucoma show a circadian IOP profile similar to normal eyes. Fluctuations of circadian IOP were greatest in secondary open-angle glaucomas as compared to other types of glaucoma.

## Figures and Tables

**Table 1:** Circadian intraocular pressure profiles in 1,408 eyes based on study groups

Time	Normal (n=336)	NPG (n=398)	OHT (n=278)	PPOAG (n=140)	POAG (n=46)	SOAG (n=210)
7 am	15.67±2.43	19.01±3.39	18.60±3.46	17.75±3.64	18.61±5.56	15.29±2.69
Noon	15.24±2.71	18.34±3.38	18.13±3.37	17.19±4.49	18.91±4.99	14.86±2.78
5 pm	14.90±2.51	18.43±3.53	17.75±3.31	17.58±3.73	19.39±5.86	14.77±2.77
9 pm	14.63±2.65	17.22±3.27	16.85±3.18	16.96±3.48	17.57±5.09	14.40±2.73
Midnight	14.08±2.76	17.55±3.37	17.19±3.34	16.82±3.64	17.76±5.35	14.60±2.73
Fluctuation	4.69±1.95	4.89±1.99	6.00±3.14	6.00±2.96	5.74±3.44	6.96±3.69

NPG, normal pressure glaucoma; OHT, ocular hypertension; PPOAG, pre-perimetric primary open-angle glaucoma; POAG, primary open-angle glaucoma; SOAG, secondary open-angle glaucoma.

**Table 2:** Number of circadian intraocular pressure profiles

Number of Profiles	Number of Eyes
1	1,408
2	863
3	532
4	328
5	211
6	117
7	68
8	8
9	4

Total	3,561

**Table 3:** Topical antiglaucoma agents administered during 1,303 intraocular pressure profiles

Medication	Number
Beta-blocker	948 (72.8%)
Dorzolamide	106 (8.2%)
Clonidine	95 (7.3%)
Brinzolamide	106 (8.2%)
Latanoprost	67 (5.1%)
Pilocarpine	42 (3.2%)
Aceclidine	32 (2.5%)
Brimonidine	11 (0.8%)
Epinephrine	2 (0.2%)
